# Ultrasonic Phased Array Testing and Identification of Multiple-Type Internal Defects in Carbon Fiber Reinforced Plastics Based on Convolutional Neural Network

**DOI:** 10.3390/ma18020318

**Published:** 2025-01-12

**Authors:** Mengyuan Ma, Zhongxin Wang, Zhihao Gao, Mingshun Jiang

**Affiliations:** School of Control Science and Engineering, Shandong University, Ji’nan 250061, China; chinamamengyuan@163.com (M.M.); wangzhongxin_13@163.com (Z.W.); gaozhihao922@163.com (Z.G.)

**Keywords:** carbon fiber reinforced plastics, convolutional neural network, multi-type defects, ultrasonic phased array testing

## Abstract

Carbon fiber reinforced plastics inevitably develop defects such as delamination, inclusions, and impacts during manufacturing and usage, which can adversely affect their performance. Ultrasonic phased array inspection is the most effective method for conducting nondestructive testing to ensure their quality. However, the diversity of defects within carbon fiber reinforced plastics makes it challenging for the current ultrasonic phased array inspection techniques to accurately identify these defects. Therefore, this paper presents a method for the ultrasonic phased array nondestructive testing and classification of various internal defects in carbon fiber reinforced plastics based on convolutional neural networks. We prepared an ultrasonic C-scan dataset containing multiple types of internal defects, analyzed the defect features in the ultrasonic C-scan images, and established an autoencoded classifier network to recognize manufacturing defects and impact defects of varying sizes. The experiments showed that the proposed method demonstrates superior defect feature extraction capabilities and can more accurately identify both impact and manufacturing defects.

## 1. Introduction

Carbon fiber reinforced plastics (CFRPs) have many advantages such as high specific strength and stiffness, corrosion resistance, and fatigue resistance [1,2,3] and have been increasingly used as substitutes for metal materials in the aerospace [3,4,5,6,7,8,9,10,11,12,13], rail transportation [7,14], automotive [4,6,8,12,13], and other industries [15]. However, due to the complexity and variety of CFRP preparation processes, it is inevitable that delamination, inclusions, holes, and other defects occur during the production process, which seriously affect the performance of CFRP products and cause safety hazards [2,3,7]. At the same time, the service process of CFRPs also produces impact damage and other defects [5,8,13,15]. Therefore, it is necessary to establish a reliable NDT system for CFRP defects to perform the accurate and efficient NDT and quality assessment of CFRP manufacturing defects.

Ultrasonic nondestructive testing has strong adaptability in composite material inspection, being especially suitable for CFRP [4,6,7,8]. Ultrasonic phased array testing technology has high detection resolution, high detection accuracy, and high sensitivity and is suitable for detecting various types of damage in carbon fiber composites with high attenuation and complex shapes [7,10,11,12,16,17]. Ultrasonic testing can be used for intelligent and automated testing to improve the efficiency, precision and reliability of testing. The following delineates the recent advancements in ultrasonic phased array testing.

Prabhakara P. et al. [18] developed a new phased array based ultrasonic drilling probe for inspecting cracks and delamination in internal concrete structures, using the computational focusing law to direct the acoustic beam to specific angles and distances, and validated the newly designed probe with CIVA software to demonstrate the feasibility of the experiment. Piao G. et al. [19] employed a phased array ultrasonic inspection method to identify the adhesive interface between composites. They proposed a classification framework based on damage indices derived from machine learning algorithms to categorize samples exhibiting three distinct adhesion conditions. Hampson R. et al. [20] proposed a hybrid simulation model of a phased array ultrasonic transducer for pressure pipeline inspection and verified the effectiveness of the model in detecting different damage types at different frequencies and numbers of array elements through a simulation model. Zhang H. et al. [16] proposed a combination of phased array ultrasonic testing and deep learning to detect and localize wrinkles in laminated composites and developed a convolutional neural network model to identify them at different depths by short-term Fourier transform of waveforms, which simplified the cumbersome analysis of low signal-to-noise ratios, non-resonance matches, and near-field effects that are often encountered in the ultrasonic inspection of composites.

Dupont-Marillia F. et al. [21] studied the application of ultrasonic phased arrays for the detection of forged steel block damage, using CIVA simulation to optimize the number of array elements of the phased array probe, the width of the array elements, as well as the ultrasonic transmission sequence and constructed a transducer based on the simulation results by experimentally showing that the defects of the reflective amplitude, the signal-to-noise ratio, and the resolution of the defects are the best. Zhao X. et al. [22] proposed an acoustic field computational model, adding directivity constraints for each array element to simulate the steering acoustic field under different directivity constraints, and the results showed that the directivity of each array element has a significant effect on the overall steering of the acoustic beam, which provides a more accurate support for the application design of phased array sensors. Puel B. et al. [23] proposed an optimization method based on evolutionary algorithms and numerical simulation for setting parameters for phased array design and optimization as well as verified the effectiveness through simulation. The array design and its delay law can effectively optimize the sound field using this method. McKee J. G. et al. [24] developed a two-dimensional phased array for experimental artificial defect imaging experiments, and the scanned multi-position images were combined to obtain whole-surface damage images. The results showed that for ultrasonically extracted surfaces, there was less error in the defect data from the real surface in order to improve the reliability of the ultrasonic testing of austenitic welds.

However, the internal defects in CFRPs are diverse, ranging from manufacturing defects, such as delamination and inclusions [2,7,25], to defects that arise during service, such as impact damage [5,8,13,15]. Although ultrasonic phased array technology can detect defects more accurately, it struggles to recognize the internal defects in CFRP for multiple types and on different scales after the defects are detected. Therefore, it is necessary to classify and recognize multiple types of internal CFRP defects on the basis of ultrasonic phased array testing.

In terms of CFRP defect identification, Zhang H. et al. [16] proposed detecting and localize wrinkles in laminated composites by combining phased array ultrasonic inspection and deep learning. A convolutional neural network model was established to recognize them at different depths with the short-term Fourier transform of waveforms. Tunukovic V. et al. [10] used an intelligent machine learning (ML) algorithm for CFRP defect detection and achieved better performance compared to amplitude thresholding and statistical thresholding techniques. However, the only CFRP defects studied by Tunukovic V. et al. were flat-bottomed holes and pre-embedded defects, not impact defects. Consequently, further research is imperative to facilitate the intelligent detection of ultrasonic phased array CFRP defects, thereby enabling the precise and expeditious identification of various types of CFRP defects.

In this paper, a defect classification and identification study based on a convolutional neural network is presented. Firstly, according to the characteristics of the various types of samples in the ultrasonic C-scan dataset, the design and construction of the convolutional neural network model were carried out. Then, the training and optimization of the model were completed by using the samples in the dataset to achieve the classification and identification of multiple specifications of data under three types of delamination defects, impact damage, and no defects in the composite materials. Finally, the comprehensive performance of the constructed network model was evaluated using the method of experimental comparison.

## 2. Sample Data and Data Enhancement

In order to study the identification of multiple defects inside composites using an ultrasonic phased array, the constructed ultrasonic C-scan dataset contains the defect data generated during both the manufacturing and use stages of composites.

### 2.1. Defective Specimen Preparation

This paper presents a study of three representative types of composite specimens: manufacturing defect specimens (manifested in the form of delamination), impact damage specimens, and defect-free specimens. The specifications, parameters, and preparation process for each are outlined below.

**Figure 1:**
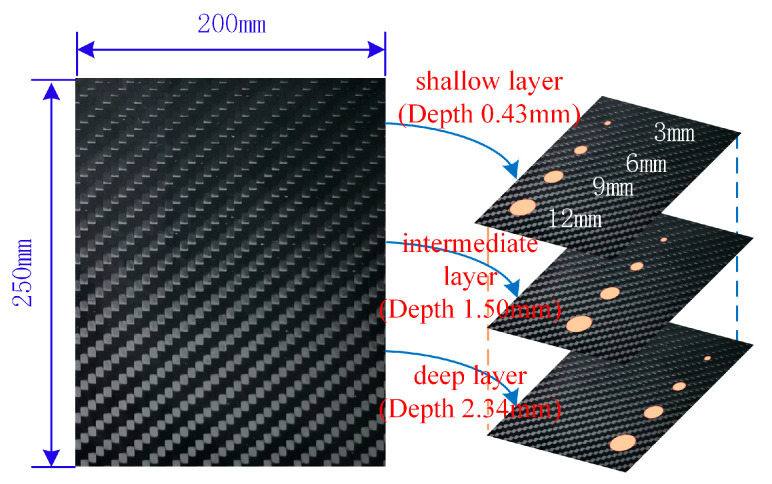
Manufacturing defective specimen.

The dimensions of the delamination defect specimen were 250×200 mm, with a thickness of 3.0 mm, as shown in Figure 1. The raw material was Toray T300 woven carbon fiber twill prepreg (FAW200/69). With a mass per unit area of 200 g/m^2^ and a nominal thickness of 0.25 mm, the specimen was laid in [90/0]7 with 14 layers, with the 0° direction aligned with the long side. Delamination defects were prepared by pre-burial of polyimide film between layers with a film thickness of 0.025 mm. The specimen layering and defect burial are illustrated in Figure 1, which depicts three pre-burial locations: a shallow layer (between layers 2 and 3), an intermediate layer (between layers 7 and 8), and a deep layer (between layers 11 and 12). Three circular defects with a diameter of 12 mm were created in the shallow layer (between layers 2 and 3), four with a diameter of 9 mm in the intermediate layer (between layers 7 and 8), and six with a diameter of 6 mm in the deep layer (between layers 11 and 12). When the polyimide film was pre-buried into the pavement layer, an appropriate amount of release agent was applied via spraying to prevent it from adhering to the prepreg pavement layer and effectively prevent it from distorting and deforming during the curing process of the specimen. The specimens were prepared using a hot press tank molding process. Following lay-up, the specimen blanks were sealed in vacuum bags and subsequently placed in the hot press tank after vacuuming. The curing temperature and pressure were 130 °C and 600 kPa, respectively.

**Figure 2:**
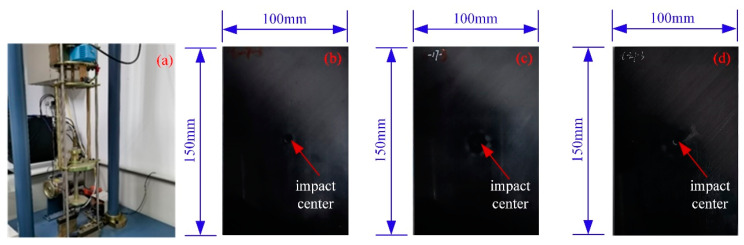
Impact test equipment and impact damage specimens. (**a**) Impact test equipment; (**b**) 7 J impact specimen; (**c**) 14 J impact specimen; (**d**) 21 J impact specimen.

The impact damage specimens selected for analysis in this study were prepared through a hammer impact test, and the apparatus utilized for this purpose is illustrated in Figure 2a. The impact specimen’s raw material was prepreg unidirectional carbon fiber (Zhongfu Shenying, T300/YH69), with a density of 1678 kg/m^3^ and an overall size of 150×100×4.5 mm. It was prepared using the hot press tank molding process, and no defective damage was observed prior to the impact test. In the drop hammer impact test, the diameter of the selected alloy steel punch was 16 mm, and the impact energy was selected to be 7 J, 17 J, and 27 J for three specifications. The impact on the composite laminate was carried out at low speed, with the impact points located near the geometric center of the sample plate. The specimens exhibiting impact damage are illustrated in Figure 2b–d.

In order to ensure the consistency of the original data samples, the defect-free specimens in this paper were chosen to be identical to the aforementioned manufacturing defects and impact damage specimens in terms of materials and specifications. Furthermore, the specimens did not contain any surface or internal defects in the carbon fiber composite material.

### 2.2. Ultrasound C-Scan Data Acquisition and Enhancement

**Figure 3:**
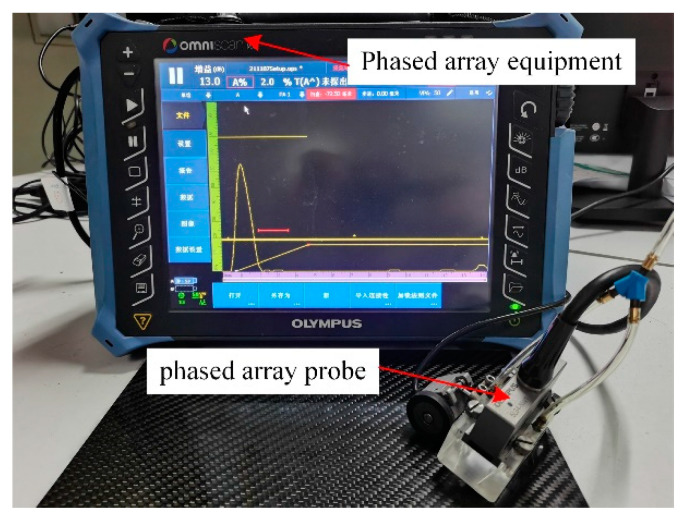
Ultrasonic phased array testing equipment.

In this paper, the ultrasonic phased array inspection equipment, illustrated in Figure 3, was employed for the acquisition of data. The host device was an Olympus portable ultrasonic phased array flaw detector, model OmniScan MX2. The phased array probe was a linear array near-wall probe, model 5L64-NM1, which consisted of six elements. The device was equipped with four arrays, with a center frequency of 5 MHz. To address the issue of near-surface blindness in ultrasonic testing, a Plexiglass wedge with a height of 20 mm was incorporated into the design. The ultrasonic inspection equipment employed pure water as the coupling agent. When ultrasonic sweeping of the aforementioned manufacturing defect specimens, impact damage specimens, and defect-free specimens was conducted, the linear focusing sweep mode was selected, the sweeping resolution was set to 1 mm, the activation aperture was set to 8, and the length of the sweep was set to 57 mm, thereby enabling a single line of sweeping to encompass a sweeping range of 57 mm × 57 mm. We used ultrasonic phased array equipment supporting software OmniPC 4.4 for ultrasonic C-scan data export.

The ultrasound C-scan image data were divided into eight groups of 75 each, for a total of 600 data points. The samples were then numbered and assigned a category code based on the type of defect present, as outlined in Table 1. In order for the network to better capture the potential differences between categories, the coding method was chosen to be one-hot encoding, the effectiveness of which has been verified [26]. The representative meanings of the defect numbers presented below align with those described in the table.

The limited quantity of initial data had a detrimental effect on the training process, leading to issues such as overfitting, poor generalization, and the problem of vanishing gradients. To address this challenge, this paper employed data enhancement techniques to augment the sample dataset. These methods encompassed a range of approaches, including flipping, rotating, and adding Gaussian noise, Gaussian blur, pretzel noise, and others.

**Figure 4:**
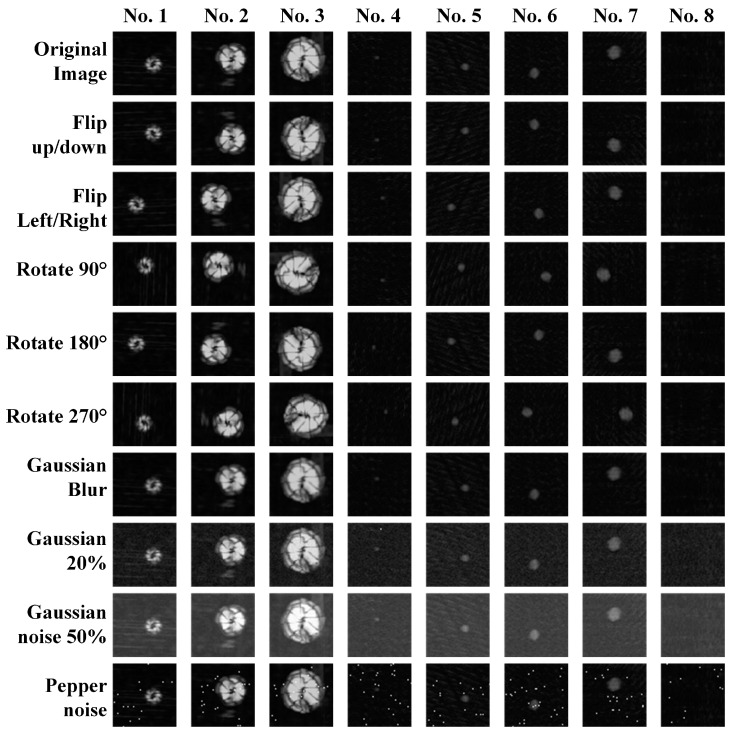
Sample enhancement.

Figure 4 illustrates the original images and the corresponding enhanced images for a variety of sample types. The addition of Gaussian noise with a mean of 20 and variance of 5, indicated by 20% Gaussian noise, and the addition of Gaussian noise with a mean of 50 and variance of 2, indicated by 50% Gaussian noise, are represented in the images. The blur radius of the Gaussian blur was set to 0.5. The mean value of the salt-and-pepper noise was set to 0, with a variance of 0.4.

Following data enhancement, the number of samples was expanded to 6000 sets, and the dataset was divided into training, validation, and testing sets according to the ratio of 7:1.5:1.5, as illustrated in Table 2. It was essential to ensure a balanced number of different sample categories in the three data sets to guarantee the optimal training and generalization performance of the network.

## 3. Classification Network Model

**Figure 5:**
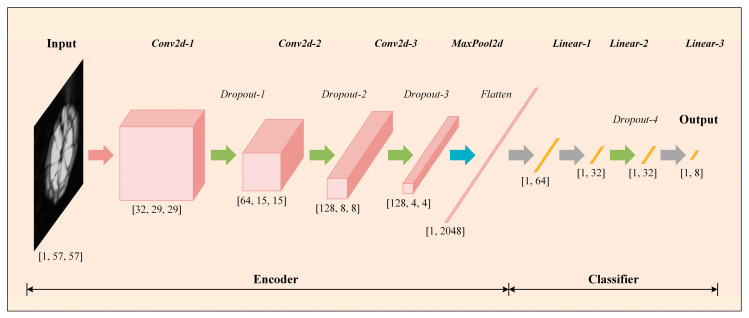
AEC net structure.

In order to achieve the efficient and high-precision classification of different classes of defects inside composite materials, this paper proposes and designs an autoencoded classifier (AEC), the network structure of which is shown in Figure 5, which contains an encoder and a classifier. The network takes the single-channel ultrasound C-scan image directly as input, which can be denoted as $I\in R^{1\times 57\times 57}$, which firstly undergoes the step-by-step two-dimensional convolution of the encoder, and then undergoes a fully connected layer, which outputs a one-dimensional feature vector with a length of 64, which can be denoted as $F\in R^{1\times 64}$. Finally, the feature vector is input to the classifier, which ultimately outputs a one-dimensional vector with a length of 8, representing the possible probability of belonging to the above 8 classes of defects. The possible probability of defects belonging to the above 8 classes can be denoted as $O\in R^{1\times 8}$. To make the dimensionality analysis clearer, the following provides a detailed step-by-step dimensionality change analysis incorporating the network structure in Figure 5:

Input layer: The input is a single-channel ultrasound C-scan image of size $I\in R^{1\times 57\times 57}$, which represents an image of 1 sample with a width and height of 57 pixels, and a channel number of 1 (grayscale image).

Encoder convolutional layers: The input image passes through several 2D convolutional layers (*Conv2d*). The function of each convolutional layer is extracting the spatial features of the image, reducing the spatial dimensions, and increasing the number of channels, resulting in a feature vector with a width and height of 8 and a channel count of 128 dimensions.

Encoder pooling layer: After the convolution operation, spatial dimensionality reduction is performed using the pooling layer. The pooling operation reduces the spatial dimensions (width and height) but maintains the number of channels of the feature vector. The output feature vector dimension after pooling is 128 × 4 × 4.

Encoder fully connected layer: The straightened pooling layer output (dimension 1 × 2048) is passed to a fully connected layer (linear), which converts the output into a one-dimensional feature vector $F\in R^{1\times 64}$ of length 64 for input to the classifier for subsequent processing.

Classifier (output layer): The feature vector F is fed into the classifier, which generates the final classification result through a number of fully connected layers and activation functions. Eventually, the network outputs a vector $O\in R^{1\times 8}$ of length 8, representing the predicted probability of defects belonging to the 8 classes.

In Figure 5, the specific operations and output dimensions are briefly labeled below each network layer. More detailed parameters can be viewed in Table 3.

The detailed structural parameters of each layer of the network are displayed in Table 3, and the specific computational procedure is as follows:

The Conv2d convolution operation and the linear fully connected layer’s computation are defined first:

(1) $$Conv2d\left(X,n\right)=relu\left(K_{n}\cdot X+b\right)$$

where Conv2d() denotes the two-dimensional convolution operation, X denotes the input to the operation, and relu is the activation function, used to introduce nonlinearity, which can improve the expressiveness of the model, defined as relu=max⁡(0,x), which converts negative numbers to zero to avoid the problem of vanishing gradients [27]. $K_{n}$ represents the n×n convolution kernel, and b is the bias weight of the convolution. ∗ denotes the convolution operation, and $K_{n}*X$ represents the convolution kernel sliding over the image region and performing the convolution computation.

(2) $$Linear\left(X\right)=W\cdot X+b$$

where Linear() denotes the fully connected layer operation, X denotes the operation input, W denotes the weight parameter, and b denotes the bias parameter. · denotes the product operation of a matrix, which is a linear operation.

(1) Encoder stage

First, the network takes image I of dimensions 1×57×57 as input after three successive Conv2d operations, and a dropout layer is added to each operation to prevent overfitting problems in the training process. Due to the small dataset, adding a dropout layer to enhance model generalization was necessary. Dropout is a simple and effective regularization method that “discards” some neurons in a neural network with a predetermined probability, i.e., sets their outputs to zero, reducing the model’s dependence on a particular neuron or feature, thus improving the model’s generalization ability [28], which is represented as follows:

(3) $$E=D\cdot Conv2d\left(D\cdot Conv2d\left(D\cdot Conv2d\left(I,3\right),3\right),3\right)$$

where I denotes the network input, E denotes the output of this process, Conv2d denotes the 2D convolution operation defined above, and D denotes the 30% randomly deactivated dropout layer.

Subsequently, the encoder intermediate output E is pooled in two dimensions and straightened into a one-dimensional vector to obtain the final output F of the encoder, and this process is represented as follows:

(4) $$F=Flatten\left(MaxPool2d\left(E\right)\right)$$

where F denotes the encoder’s encoded output, MaxPool2d denotes two-dimensional pooling, and Flatten denotes the straightening operation.

(2) Classifier stage

The encoder output F is computed through two consecutive Linear fully connected layers to obtain the final defect category probability prediction output; this process is represented as follows:

(5) $$O=Linear\left(D\cdot Linear\left(F\right)\right)$$

where O denotes the class probability output of the network, Linear denotes the fully connected layer operation described above, and D denotes the dropout layer with 30% random deactivation.

### 3.1. Loss Function

In classification tasks, the use of a mean square error loss function is a common practice for effectively guiding neural network learning and improving classification accuracy. Mean squared error (MSE) [29], a widely utilized loss function in regression and classification problems, assesses the model’s prediction error by calculating the mean of the squared differences between the predicted and true values. The formula for its calculation is as follows:

(6) $$Loss=MSE=\frac{1}{n}\sum_{i=1}^{n}{\left(y_{i}-{\hat{y}}_{i}\right)}^{2}$$

where $y_{i}$ denotes the label value in the form of the unique hot code, ${\hat{y}}_{i}\in \left[0,1\right]$ denotes the probability value that the model predicts to be the ith class, and n is the number of training batches, where the smaller the value of the mean square error loss function, the closer the prediction result is to the true value. By minimizing the mean square error loss, the classification prediction of the model can be made closer to the true label value, which in turn improves the classification performance of the model.

### 3.2. Training Hyperparameters

Table 4 enumerates the training hyperparameter settings utilized for the AEC model in this paper. These comprise Learning rate, epoch, batch size, optimizer, and learning rate scheduler. The specific parameters are as follows: the learning rate was set to 0.001, the number of training rounds was 500, and the batch size was 16. The optimizer employed the Adam algorithm to achieve a balance between convergence speed and stability. The learning rate scheduling strategy was step LR, which decayed the learning rate by a factor of 0.8 every 50 rounds, thereby further improving the convergence effect and performance stability of the model. The reasonable setting of these parameters ensures the efficient training of the model while effectively avoiding overfitting.

### 3.3. Training Environment

Table 5 enumerates the hardware and software environment configurations that the network training program relies on to ensure the reproducibility of the experiments and the efficiency of model training. The training environment included the operating system, CPU, GPU, memory, and relevant deep learning framework and tool version information.

### 3.4. Evaluation Indicators

In order to judge the strengths and weaknesses of the network model (classification performance), this paper used the following evaluation metric commonly used for classification models: accuracy (Acc). This metric is a key measure of a model’s classification performance on the unseen test set. 

Acc represents the classification accuracy of the model on an unseen test set, which is calculated as

(7) $$Acc=\frac{P_{r}}{N}$$

where N denotes the total number of samples, and $P_{r}$ denotes the number of samples for which the network model predicts the correct outcome (classification). Acc takes values between [0,1], with larger values indicating stronger overall classification performance of the model.

## 4. Test Results

In order to facilitate a comparative and analytical assessment of the performance advantages and disadvantages of AEC networks, this paper selected the most representative state-of-the-art network models in the field of image classification, namely, AlexNet [30] and ShuffleNet-V2 [31], for simultaneous training. The three network models were trained using the same dataset and training hyperparameters throughout the training process. Ultimately, 500 rounds were trained with the identical loss function with the same hardware and software environment. The details are consistent with the descriptions in the aforementioned sections and are not repeated here.

**Figure 6:**
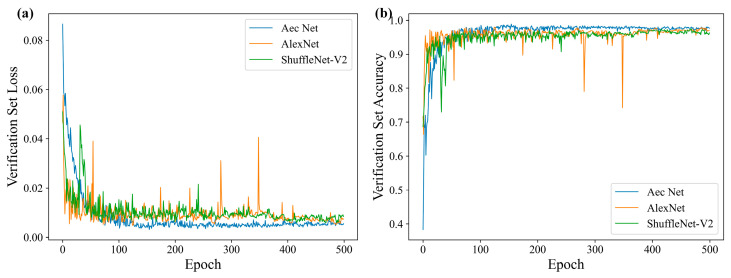
Model training curves. (**a**) Loss curve; (**b**) accuracy curve.

Figure 6a illustrates the loss changes of each model on the validation set throughout the training process. It is evident that all three models converged, albeit with varying degrees of oscillation and smoothing. AlexNet exhibited the most pronounced oscillation, and there were instances where the loss function experienced a sudden increase. However, the AEC network loss changes were the most gradual, and, with a gradual decline in the training process, they eventually converged. This indicated that the network structure was set up reasonably and that there was no overfitting problem in the training process.

Figure 6b depicts the evolution of accuracy for each model on the validation set throughout the training process. Table 6 presents the final accuracy of each model, the highest accuracy attained during training, the mean accuracy across 300–500 training rounds, and the number of training rounds where the highest accuracy was achieved. It is evident that the AEC network attained the highest accuracy rate of 97.69% at the conclusion of training, 98.89% at the peak accuracy rate, and 97.74% at the mean accuracy rate of 300–500 rounds. This signified that the network exhibited superior classification performance in comparison to conventional image classification networks.

Meanwhile, the number of trainable parameters, the time required for training, and the memory consumption of the model during computation (here, memory consumption refers to the memory consumption of the computation process when a single image is fed into the network) are quantitatively compared for each model in Table 6. In this paper, the number of trainable parameters of the AEC network was reduced by a factor of 100 compared to AlexNet and by a factor of 5 compared to ShuffleNet-V2, which had the least training time and memory consumption. It should be noted that the issue of computational time consumption arose due to the fact that ShuffleNet-V2 calls a significant number of external functions within the model structure.

In conclusion, the AEC network, with its straightforward and logical structural design, exhibits a lightweight characteristic, and the memory consumption and the number of trainable parameters are significantly reduced in comparison to the traditional image classification network, while the network’s performance and classification accuracy are maintained.

## 5. Analysis and Discussion

In order to further validate the generalization performance of the networks and to analyze the feature extraction capability of the networks, classification was performed on the test set (completely uninvolved in the training process) using the model parameters at the end of the training and using the model parameters at the time when the highest accuracy occurred. The classification accuracies are shown in Table 7. It can be seen that all three models showed superior performance on the unseen data set, and, in both cases, AEC had the highest classification accuracy, indicating that the network had optimal generalization capabilities.

As the loss and accuracy values remained in an oscillatory state at the point of maximum accuracy for both AlexNet and AEC Net, the model had not yet reached a fully converged state. Consequently, the model at the conclusion of the training period was selected for the subsequent analysis of its feature extraction capabilities. The model that underwent 500 rounds of training was expected to demonstrate superior convergence and generalization abilities, as well as a more comprehensive coverage of the data feature distribution.

**Figure 7:**
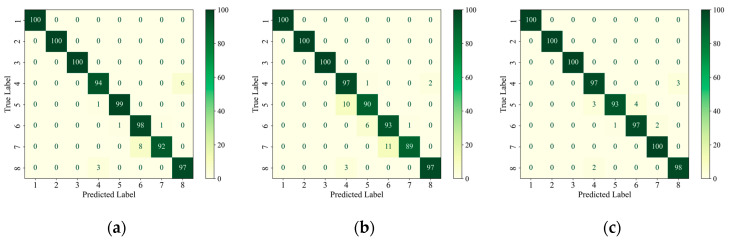
Confusion matrix of model classification results on the test set. (**a**) AlexNet; (**b**) ShuffleNet-V2; (**c**) AEC Net.

**Figure 8:**
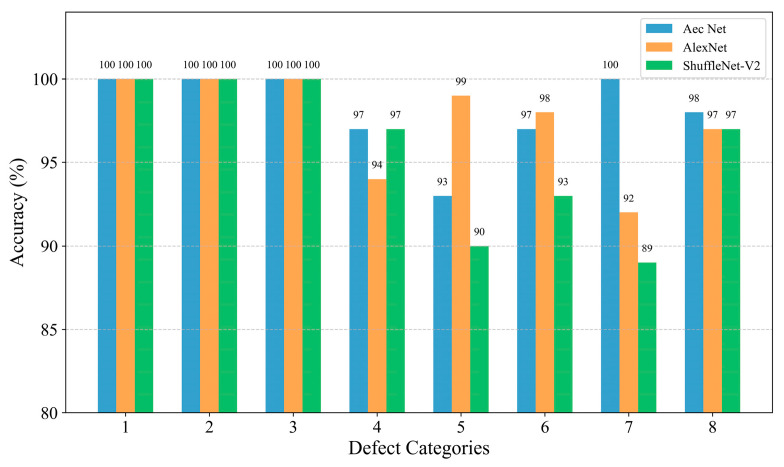
Histogram of model classification results on the test set.

In order to further analyze the network’s ability to extract features from data with different types of defects, firstly, the confusion matrix was drawn based on the classification of each type of defect in the test set, as shown in Figure 7. The confusion matrix visualizes the model’s predictions on different categories and is a commonly used tool for evaluating the performance of classification models. The core idea is to compare the true labels with the predicted labels and generate a matrix where each element of the matrix represents the model’s prediction on a specific category [32]. Given that the AEC network was utilized for the identification and classification of defects, the implementation of statistical analysis through linear regression [33] was rendered unnecessary. The classification accuracy of each type of defect is plotted as a histogram based on the confusion matrix, as shown in Figure 8, and is quantified in Table 8.

**Figure 9:**
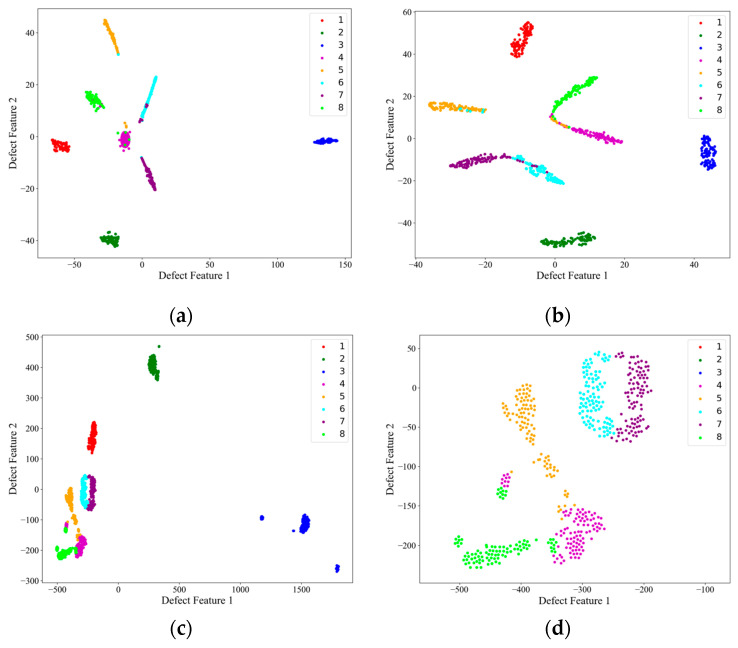
PCA downscaling of model output features on the test set. (**a**) AlexNet; (**b**) ShuffleNet-V2; (**c**) AEC net; (**d**) AEC net local details.

Then, on the test set, the image features extracted from the encoder structure of each network, i.e., the encoder output F described above, were subjected to PCA downscaling and visualized, and the results are shown in Figure 9. Figure 9d shows a local zoom of the red-boxed region in the PCA feature downscaling plot of the AEC network. Combined with the confusion matrix in Figure 7, it can be seen from the feature downscaling plots that all three networks had the strongest feature extraction ability for defects 1, 2, and 3, which corresponded to the red, green, and blue colors, respectively, and the feature boundaries were completely independent from each other and from the other classes of defects. For AlexNet and ShuffleNet-V2, there was a strong coupling between the features of the other classes of defects, and, from Figure 9d, it can be seen that the AEC network was more capable of distinguishing the features of the other defects and had the lowest coupling, despite the fact that the defect feature boundaries were connected.

Although the benchmark dataset established in this paper and the proposed AEC classification method were able to successfully perform the classification of internal defects in composites in most of the cases, there were still some cases where lower accuracy and defect confusion occurred. From the above analysis, it can be seen that there are two main problems with the method: (1) the identification of small-size defects (e.g., the classification accuracies of category 4 and category 5, 3 mm and 6 mm delamination defects, on the test set were only 97% and 93%, which are lower than the average value of 98.875%), and (2) the confusion problem of similar defect features (e.g., from Figure 9d, it can be seen that the feature boundaries of the AEC-extracted defects of categories 4, 5, 6, 7, and 8 are adjacent and have no obvious boundaries, although they are relatively independent).

In our study, although the proposed methodology performed well in most cases, there are were some failure cases in some specific scenarios. Some of the failure cases are analyzed below.

**Figure 10:**
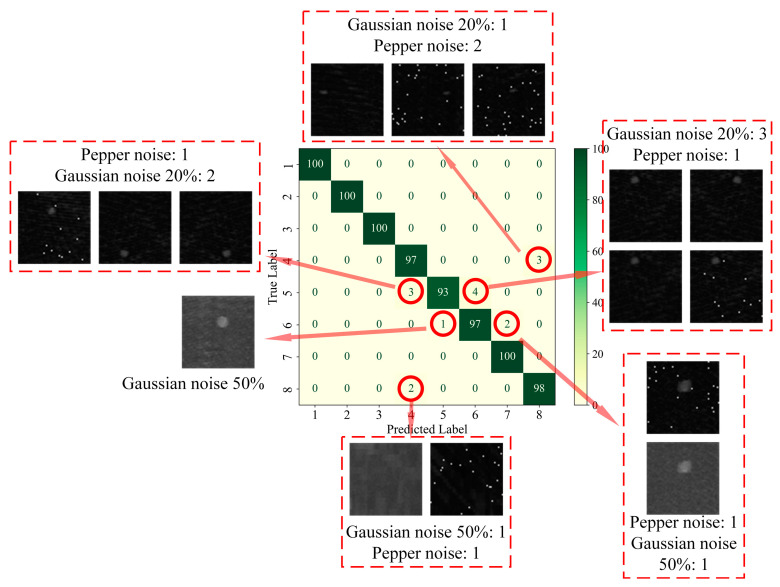
Failure cases on the test set.

As can be seen in Figure 7c, on the test set, category 4 (3 mm delamination defects) was identified with three fault cases, all of which were misclassified as no defects; category 5 (6 mm delamination defects) was identified with seven fault cases, three of which were misclassified as category 4, and the other four were misclassified as category 6 (9 mm delamination defects); category 6 was identified with three fault cases, one of which was misclassified as category 5 and the other two were misclassified as category 7 (12 mm delamination defects); category 8 (no defects) was identified with three failure cases, all of which were misclassified as category 4, as shown in Figure 10.

Figure 10 shows all 15 fault cases on the test set. It can be found that all the fault cases were obtained by sample enhancement, of which 6 were obtained by adding Gaussian noise 20%, 3 were obtained by adding Gaussian noise 50%, and 6 were obtained by adding salt-and-pepper noise.

We believe that there are three main reasons for these two problems: (1) the small size of the benchmark dataset, (2) the high sensitivity of AEC to noise, and (3) the limited feature extraction ability of AEC of similar defects.

Due to the experimental conditions and workload problems, the number of benchmark datasets collected in this study was small, which may have led to the problem of poor generalization ability of the network model, and the samples with unseen defect types may have experienced a decrease in recognition ability. Although this paper expands the benchmark dataset by means of data enhancement, the enhancement process had errors and could not represent the real sample distribution. In the future, we will further expand the benchmark dataset, including sample type and number. In addition to the influence of the dataset, the limited sensitivity to noise and feature extraction ability of AEC networks are also possible reasons. For similar defect types, the feature extraction capability of traditional CNNs may be insufficient to accurately capture multi-level structural information. Subsequent research efforts may consider adopting more powerful network structures (e.g., Transformer ViT, graph neural networks) in order to enhance the feature extraction and generalization capabilities of the network.

In summary, the AEC network still had the optimal feature extraction ability given its lightweight characteristic and has better generalization ability for unseen data. The AEC network had 100% differentiation ability for impact defect features, and there was weak confusion for the 3 mm, 6 mm, and 9 mm layered defect feature boundaries. This can be attempted to be resolved in subsequent studies by expanding the sample set or by enhancing the defect features through effective preprocessing means.

## 6. Conclusions

In order to address the issue of defect classification and identification in the context of ultrasonic inspection of carbon fiber composites, we constructed an ultrasonic C-scan dataset of composites. Based on this dataset, we developed an autoencoded classifier and a method for the classification and identification of defects in composites using a convolutional neural network. This method is capable of classifying and identifying delamination defects of varying sizes, impact damages of differing energies, and defect-free samples of composites under three distinct categories.

The designed AEC network has a simple and reasonable structural design and is lightweight to guarantee network performance and classification accuracy. In comparison to traditional image classification networks, the memory consumption and the number of trainable parameters are significantly reduced, thereby improving the operation speed and computational efficiency of the classifier. The designed AEC network, despite its lightweight characteristics, exhibits robust feature extraction capabilities and superior generalization abilities with unseen data. The model demonstrates enhanced overall adaptability and holds considerable promise for practical applications.

## Figures and Tables

**Table 1:** Sample data distribution and numbering.

Defect Type		Number	Number of Samples	Number of Expansion Samples	Category Code
Defect-free	-	8	75	750	10000000
Delamination defects	12 mm	7	75	750	01000000
	9 mm	6	75	750	00100000
	6 mm	5	75	750	00010000
	3 mm	4	75	750	00001000
Impact Defects	27 J	3	75	750	00000100
	17 J	2	75	750	00000010
	7 J	1	75	750	00000001
Total	-	-	600	6000	-

**Table 2:** Division of the dataset.

Total Number of Samples	Training Set	Validation Set	Test Set
6000	4400	800	800

**Table 3:** AEC network structure parameters.

	Layer	Output Shape	Convolutional Kernel	Stepper	Padding	Activation Function	Number of Trainable Parameters
Encoder	Conv2d-1	[−1, 32, 29, 29]	3	2	1	relu	320
	Dropout-1	[−1, 32, 29, 29]	-	-	-	-	0
	Conv2d-2	[–1, 64, 15, 15]	3	2	1	relu	18,496
	Dropout-2	[−1, 64, 15, 15]	-	-	-	-	0
	Conv2d-3	[−1, 128, 8, 8]	3	2	1	relu	73,856
	Dropout-3	[−1, 128, 8, 8]	-	-	-	-	0
	MaxPool2d	[−1, 128, 4, 4]	2	1	-	-	0
	Flatten	[−1, 2048]	-	-	-	-	0
	Linear-1	[−1, 64]	-	-	-	-	131,136
Classifier	Linear-2	[−1, 32]	-	-	-	relu	2080
	Dropout-4	[−1, 32]	-	-	-	-	0
	Linear-3	[−1, 8]	-	-	-	sigmoid	264

**Table 4:** Training hyperparameters.

Learning Rate	Epoch	Batch size	Optimizer	Scheduler
0.001	500	16	Adam	Step LR (50, 0.8)

**Table 5:** Network training environment.

Name	Value
System	Ubuntu 20.04.4 LTS
CPU	Intel(R) Xeon(R) Gold 6248R CPU @ 3.00 GHz (Intel, Santa Clara, CA, USA)
GPU	NVIDIA GeForce RTX 3090 (NVIDIA, Santa Clara, CA, USA)
Memory	24 GB
Torch Version	1.8.1 + cu111
CUDA Version	11.6

**Table 6:** Comparison of model training results on the validation set.

Model	AlexNet	ShuffleNet-V2	AEC Net
Number of trainable parameters	23,466,696	1,261,372	226,152
Training time (s)	2308.1	10461.1	1808.5
Memory consumption (MB)	90.05	8.60	1.65
Accuracy at the end of training (%)	96.92	96.23	97.69
Maximum accuracy rate (%)	98.03	97.52	98.89
Average accuracy rate of 300–500 rounds (%)	96.45	96.29	97.74
Highest accuracy rate appeared rounds	125	468	154

**Table 7:** Model classification accuracy on the test set.

Model	Alex Net	ShuffleNet-V2	AEC Net
At end of training (%)	97.50	95.75	98.125
At highest accuracy (%)	97.375	97	98.875

**Table 8:** Classification accuracy of defects by category.

Type of Defect	AlexNet Acc (%)	ShuffleNet-V2 Acc (%)	AEC Net Acc (%)
1	100	100	100
2	100	100	100
3	100	100	100
4	94	97	97
5	99	90	93
6	98	93	97
7	92	89	100
8	97	97	98

## Data Availability

Data is contained within the article.
